# Fluid-like cathode enhances valuable biomass production from brewery wastewater in purple phototrophic bacteria

**DOI:** 10.3389/fmicb.2023.1115956

**Published:** 2023-03-13

**Authors:** Carlos Manchon, Yeray Asensio, Fernando Muniesa-Merino, María Llorente, Álvaro Pun, Abraham Esteve-Núñez

**Affiliations:** ^1^Universidad de Alcalá, Alcalá de Henares, Madrid, Spain; ^2^Nanoelectra, Alcalá de Henares, Madrid, Spain; ^3^IMDEA WATER, Alcalá de Henares, Madrid, Spain

**Keywords:** purple phototrophic bacteria, electroactive, cathode, biorefinery, wastewater, biomass, fluidized, fluid-like

## Abstract

The climate crisis requires rethinking wastewater treatment to recover resources, such as nutrients and energy. In this scenario, purple phototrophic bacteria (PPB), the most versatile microorganisms on earth, are a promising alternative to transform the wastewater treatment plant concept into a biorefinery model by producing valuable protein-enriched biomass. PPB are capable of interacting with electrodes, exchanging electrons with electrically conductive materials. In this work, we have explored for mobile-bed (either stirred or fluidized) cathodes to maximize biomass production. For this purpose, stirred-electrode reactors were operated with low-reduced (3.5 e^−^/C) and high-reduced (5.9 e^−^/C) wastewater under cathodic polarization (−0.4 V and –0.8 V vs. Ag/AgCl). We observed that cathodic polarization and IR irradiation can play a key role in microbial and phenotypic selection, promoting (at –0.4 V) or minimizing (at –0.8 V) the presence of PPB. Then, we further study how cathodic polarization modulates PPB biomass production providing a fluid-like electrode as part of a so-called photo microbial electrochemical fluidized-bed reactor (photoME-FBR). Our results revealed the impact of reduction status of carbon source in wastewater to select the PPB photoheterotrophic community and how electrodes drive microbial population shifts depending on the reduction status of such carbon source.

## 1. Introduction

For decades, the strategy to deal with wastewater was aimed at the removal of contaminants. However, nutrients from urban and industrial wastewater represent a valuable source of resources (Hülsen et al., 2016; Puyol et al., 2017; Capson-Tojo et al., 2020). This is how the concept of biofactory was born, in which wastewater treatment plants are upgraded to facilities in which nutrients are recovered from wastewater, greatly improving in economic and environmental terms (Verstraete et al., 2009).

One of the most environmentally friendly and cost-effective approaches for nutrient recovery is their biological accumulation (Batstone et al., 2015). Specifically, purple phototrophic bacteria (PPB) have been found to be remarkably efficient in recovering nutrients compared to other phototrophic microbes such as algae and cyanobacteria (Puyol et al., 2017; Delamare-Deboutteville et al., 2019). In the framework of nutrient recovery, PPB are used photoheterotrophically where they use infrared light as main energy source and a wide range of organic compounds (Capson-Tojo et al., 2020).

Since the discovery of PPB, researchers have deeply explored the metabolic diversity that allows them to be so ubiquitous (Madigan and Jung, 2009). One of the fundamental pillars of its versatility is the ability to maintain redox homeostasis through the so-called “electron sinks” (McKinlay and Harwood, 2010). These metabolic pathways such as carbon fixation or nitrogen production consume the excess reducing power, ultimately giving rise to other processes such as synthesis of biomass (proteins) or PHA (Bayon-Vicente et al., 2021; Montiel-Corona and Buitrón, 2021).

The activation of the “electron sink” pathways depends mainly on degree of reduction for the carbon source. In short, those carbon sources more reduced than biomass (4.5 e^−^/C) require the activation of “electron sink” pathways (McKinlay and Harwood, 2010; Vasiliadou et al., 2018).

Therefore, the optimal growth of PPB requires the control of electron flows by means of artificial addition of electrons to drive metabolism toward different targeted bioproducts (Varfolomeyev, 1992; Vasiliadou et al., 2018). One of the approaches is electrochemistry-based, in which electrodes are used to provide electron acceptors or donors to electroactive microorganism (Lovley and Holmes, 2022). Since the first interaction between a purple phototrophic bacterium and an electrode was reported (Bose et al., 2014), researchers have exploited its potential (Venkidusamy and Megharaj, 2016; Vasiliadou et al., 2018; Soundararajan et al., 2019; Guardia et al., 2020). These discoveries have demonstrated the malleability of PPB metabolism under the control of an electrode, increasing the biomass production and improving nutrient removal (Guzman et al., 2019; Edreira et al., 2021; Manchon et al., 2023a).

In contrast with conventional electrode-based cultivation of electroactive bacteria (rods, felt, plates) the use of fluid-like electrodes has been demonstrated for growing electroactive planktonic cells (Tejedor-Sanz et al., 2018). Actually, fluid-like anodes can be used for treating brewery wastewater with both non-photosynthetic bacteria (Tejedor-Sanz et al., 2020; Asensio et al., 2021) and PPB (Manchon et al., 2023b) as part of a photo-electrofermentation process where pollutant removal is maximized while a valuable product is recovered: the biomass of PPB.

In addition to exhibit an anodic role, fluid-like electrodes may act as electron donor (cathode) in electron uptake processes so microorganism can perform reductive metabolism like denitrification or CO_2_ fixation (Tejedor-Sanz et al., 2020; Llorente et al., 2023). In this work, we have explored for first time the role of fluid-like cathodes to stimulate the “electron sink” mechanism from PPB in order to maximize nutrient recovery from brewery wastewater treatment. Furthermore, we have explored how the degree of reduction in carbon sources may impact on PPB electrofermentation.

## 2. Materials and methods

### 2.1. Microbial inoculum and microbial growth

As inoculum (1%) for all experiments, we used a microbial consortium selected from an phtotoelectrochemical fluid-like reactor operated under anodic polarization (0.2 V vs. Ag/AgCl (Manchon et al., 2023b). The cultivation of PPB was performed under continuous IR-illuminated conditions (850 nm).

### 2.2. Experimental set-up

#### 2.2.1. Screening brewery wastewater photo-electrofermentation

Photo-electrofermentation screening was performed in 250 ml single-chamber reactors fed with wastewater from the Mahou San Miguel brewery (Alovera, Spain). In order to evaluate the effect of reduction status on PPB culture, we used two different brewery wastewater: high-reduced wastewater (5.9 e^−^/C) and low-reduced wastewater (3.5 e^−^/C).

**Figure 1:**
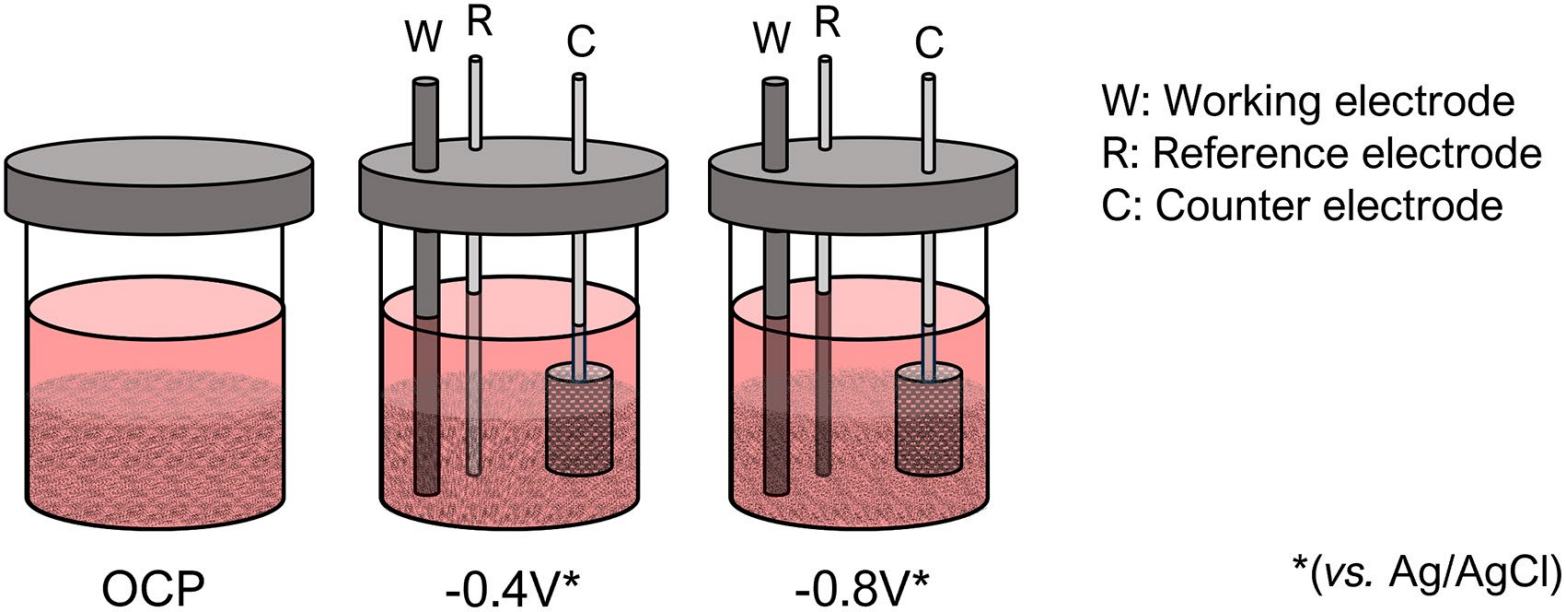
Scheme of the experimental set-up used for the photo-electrofermentation screening with brewery wastewater.

In order to explore the effect of polarization on PPB cultivation, different treatments were compared: Open Circuit Potential (OCP), −0.4 V (vs. Ag/AgCl) and –0.8 V (vs. Ag/AgCl; Figure 1).

The polarized reactors (−0.4 V and –0.8 V) were made up of three electrodes: a working electrode (WE) whose electrical potential is controlled by a potentiostat with respect to a reference electrode (RE, 3 M KCl Ag/AgCl; Hanna Instruments S.L.) and a counter electrode (CE) in which the counterreaction takes place. In these systems, the working electrode was made up of a stirred bed of vitreous carbon (Sigradur G, HTW, Germany), using a carbon rod (Mersen, Courbevoie, France) as current collector (Liu et al., 2014; Caizán-Juanarena et al., 2020). Moreover, a platinized titanium mesh (Inagasa S.A., Barcelona, Spain) was used as counter electrode. The bed was stirred with a magnetic bar. Electrochemical measurements were performed with a multipotentiostat NEV6 (Nanoelectra S.L., Alcalá de Henares, Spain) and electric current was measured with a Keithley Series 6,400 multimeter (Tektronix/Keithley, Cleveland, United States). Additionally, a non-polarized reactor without electrically conductive material (Electrode-free) was operated as a control.

#### 2.2.2. Giving insight into photo-electrofermentation using photo microbial electrochemical fluidized-bed reactor: Photo microbial electrochemical fluidized-bed reactor

Photo-electrofermentation in presence of a fluid-like cathode was performed in microbial electrochemical fluid-like cathode reactors (Tejedor-Sanz et al., 2020) fed with synthetic wastewater containing NaHCO_3_ 2.5 g L^−1^, NH_4_Cl 0.5 g L^−1^, NaH_2_PO_4_.2·H_2_O 0.41 g L^−1^, KCl 0.1 g L^−1^, a mixed of vitamins 10 ml/l, a mixed of minerals 10 ml/l. Medium was sparged with N_2_:CO_2_ (80:20) to remove dissolved oxygen. We tested two organic acids as carbon source to a final concentration of 1 g·L^−1^ of total organic carbon (TOC): malate (3 e^−^/C) and acetate (4 e^−^/C).

**Figure 2:**
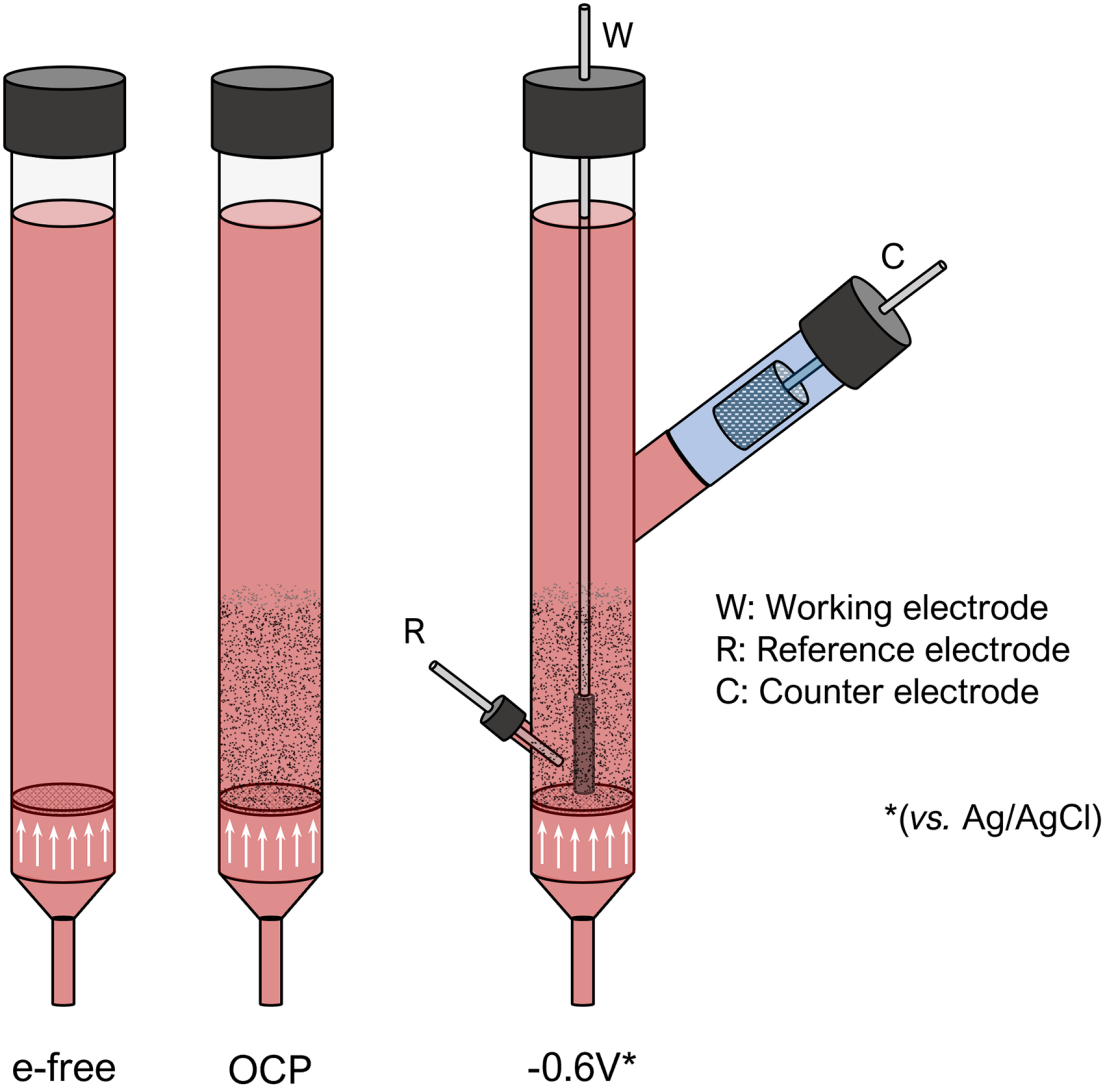
Scheme of the experimental set-up used for the fluid-cathode photo-electrofermentation.

Separation of anodic and cathodic is key in electrofermentation so we designed and built a bicameral ME-FBR (Figure 2). Thus, we operated or ME-FBR, with (a) non-polarized electroconductive fluid-like bed (OCP); (b) electroconductive fluid-like bed polarized at −0.6 V (vs. Ag/AgCl); and (c) an electrode free system as control. The polarized reactor potential was –0.6 V to ensure cathodic polarization throughout the bed.

The reactors, made of borosilicate, had an inlet at the bottom and an outlet at the top to recirculate the liquid upward. At the bottom of the reactor, a fritted plate was used as a diffuser. In the polarized reactor, in addition to the main chamber, a second chamber separated by an ion exchange membrane, was designed, to house the counter electrode.

We used a multichannel peristaltic pump (Dinko, Barcelona, Spain) to recirculate the liquid in all the reactors. In the reactors with electroconductive bed both OCP and polarized at –0.6 V) vitreous carbon particles (Sigradur G, HTW, Germany) were used as bed. In the polarized reactor, following the same three-electrode scheme explained in the previous section, a current collector (Mersen, Courbevoie, France) was used to polarize the working electrode (fluidized bed). A reference electrode (Hanna Instruments S.L.) was placed immersed in the bed. A platinized titanium mesh (Inagasa S.A., Barcelona, Spain) was placed in the anodic chamber as a counter electrode. The ion exchange membrane between the chambers was provided by Nafion (The Chemours Company FC, LLC).

### 2.3. Experimental design

#### 2.3.1. Screening photo-electrofermentation in brewery wastewater

Stirred reactors were operated under IR-illuminated conditions in fed-batch with a hydraulic retention time (HRT) of 5 days during a period enough to reach steady state. After 1 week of operation under steady state, samples were taken to assess biomass production and analyze microbial populations.

The OCP acted as non-artificially polarized control. We used two cathodic potentials: −0.4 V and –0.8 V (vs. Ag/AgCl).

#### 2.3.2. Giving insight into fluid-like cathodic photo-electrofermentation

Fluid-like bed reactors were operated under IR-illuminated (13 W/m^2^) conditions in batch. The systems were operated until the stationary phase of growth was reached. At the end of the experiment, samples were taken to assess biomass production and analyze microbial populations.

### 2.4. Analytical methods

Samples were filtered (0.22 micrometer) and Total Organic Carbon (TOC) and Chemical Oxygen Demand (COD) were measured. COD was quantified using a commercial kit (Merck Millipore, Germany) as previously described (Asensio et al., 2021). TOC was measured by a TOC-VCSH Shimadzu analyzer. Organic acids concentration (malate and acetate) were quantified by HPLC (HP series 1,100, UV detector 210 nm and Supelco C-610H column). Inorganic nitrogen compounds were analyzed by ionic chromatography (Metrohm 930 Compact Ion Chromatograph Flex), for which they were filtered at 0.45 μm and later at 0.22 μm with a tangential filter.

## 3. Results and discussion

Inspired by the performance of fluidized anodes for the metabolic control of PPB (Manchon et al., 2023b), in this work, we have explored the role of cathodes in the treatment and recovery of nutrients from industrial brewery wastewater. After screening the potential of stirred cathodes in single-chamber reactors (section 3.1), we have delved into the fluid-like cathode performance using a new bichambered design (section 3.2).

### 3.1. Screening photo-electrofermentation for treating brewery wastewater

Stirred reactors with electrically conductive granular material were operated with brewery wastewater under IR-illuminated conditions to promote PPB growth. We validated the performance of non-externally polarized reactor (OCP) versus reactors whose stirring electrode bed were polarized at –0.4 V (vs. Ag/AgCl) to promote direct extracellular electron uptake and at –0.8 V (vs. Ag/AgCl) to promote hydrogen-mediated extracellular electron uptake.

As previously reported (McKinlay and Harwood, 2010), the reduction status of carbon source present in wastewater is key for PPB metabolism. Thus, the reactors were fed with wastewater showing different reduction states: high reduction status (5.9 e^−^/C) and low reduction status (3.5 e^−^/C; Table 1).

**Table 1:** Water quality parameters of the two type of brewery wastewater used in the screening.

Wastewater	COD [ppm]	TOC [ppm]	TN [ppm]	TOC:COD	e^−^/C
High-reduced	232	47	9.9	0.20	5.9
Low-reduced	886	391	30.3	0.44	3.5

The e^−^/C ratio was calculated based on the TOC: COD ratio as shown in Supplementary material.

#### 3.1.1. Cathodic polarization does not dramatically affect the treatment performance of brewery wastewater

**Figure 3:**
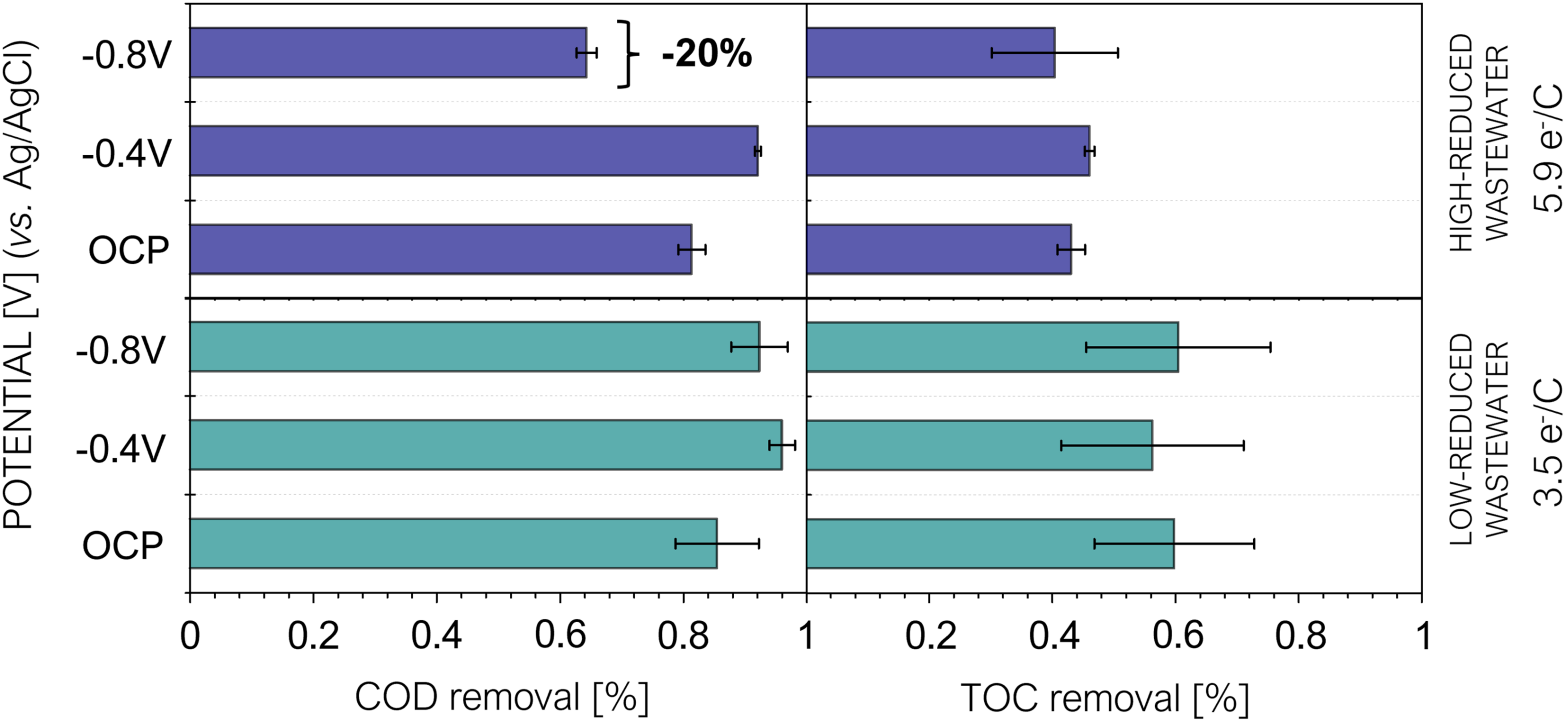
Chemical oxygen demand (COD) and total organic carbon (TOC) removals ± standard error under open circuit potential (OCP), −0.4 V and −0.8 V. HRT = 5 days. Purple bars correspond to reactors fed with high-reduced wastewater and blue bars corresponds to the reactors fed with low-reduced wastewater.

Water quality analysis showed similar COD removal in all treatments with both types of tested wastewater (Figure 3). Therefore, cathodic polarization did not exert a dramatic effect in organic pollutants removal, except for the apparent lower removal (−20%) in the high-reduced wastewater (5.9 e^−^/C) under −0.8 V (vs. Ag/AgCl) polarization.

The complexity of the compounds in the brewery wastewater, such as sulfur compounds or some metals, may hinder the evaluation of the organic matter removal by COD analysis (Watts and Dean Adams, 1983). The analysis of total organic carbon (TOC), on the other hand, allowed a more accurate quantification of organic matter. Our TOC results revealed that the different COD removal observed in the high-reduced wastewater does not show a negative impact in the biodegradation performance (Figure 3). We hypothesize that the potential –0.8 V vs. Ag/AgCl could have favored electrofermentation processes (Prévoteau et al., 2020), giving rise to the transformation of the organics into other more reduced compounds. Although this finding requires further exploration, it was observed under hydrogen-mediated conditions (−0.8 V) and not through direct extracellular electron uptake (−0.4 V).

Nitrogen removal showed highly variable results throughout the assays with no significant impact of polarization (Supplementary Figure S1).

#### 3.1.2. Cathodic polarization effectively promotes biomass production

**Figure 4:**
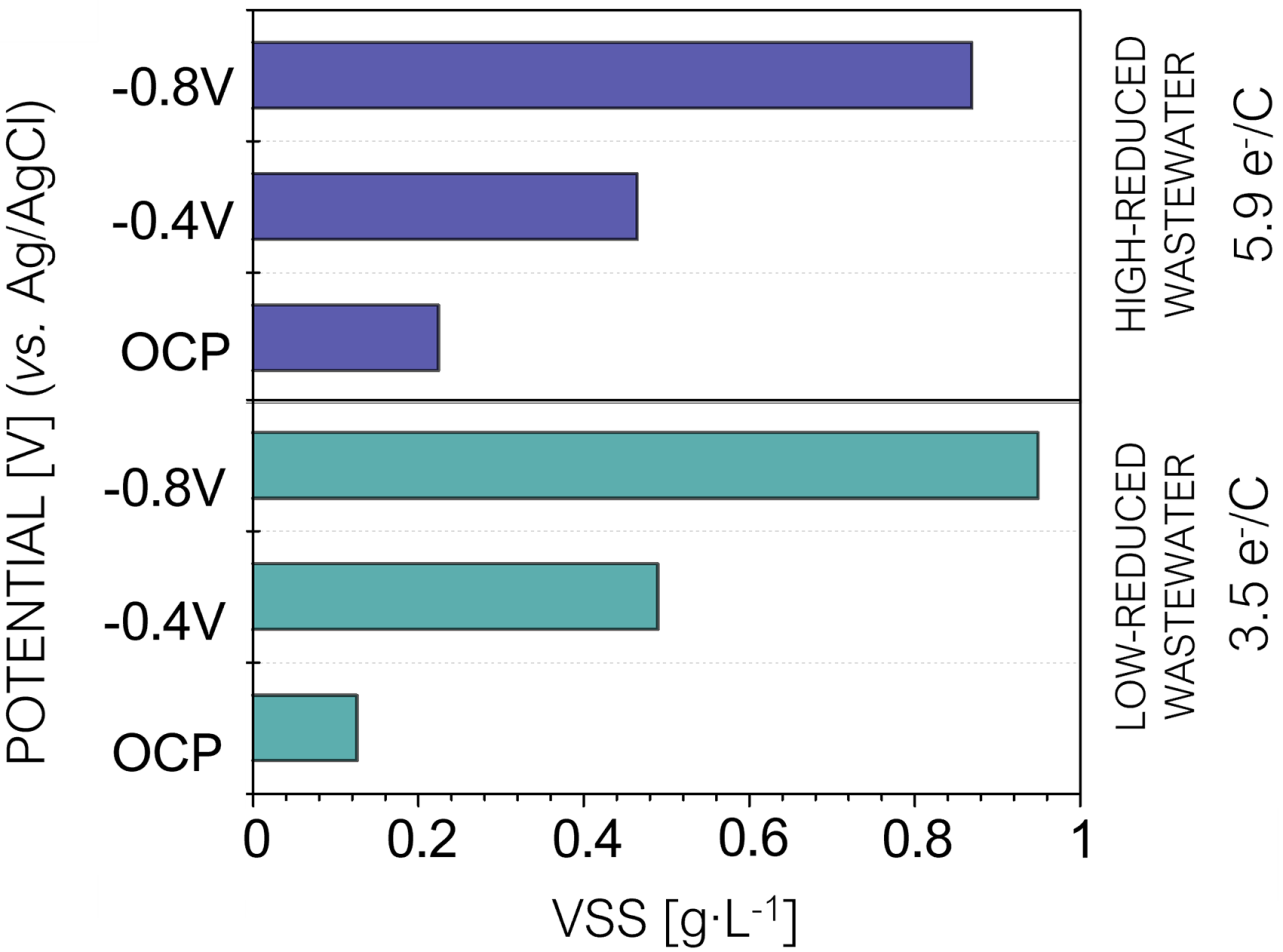
Volatile suspended solids (VVS) produced at the end of the experiment under open circuit potential (OCP), −0.4 V and −0.8 V. HRT = 5 days. Purple bars correspond to reactors fed with high-wastewater and blue bars corresponds to the reactors fed with low-reduced wastewater.

Extracellular electron uptake from a cathode is strongly connected to carbon dioxide fixation in PPB (Guzman et al., 2019). Therefore, we hypothesized that providing extra electrons to PPB *via* a cathode in brewery wastewater treatment could maximize carbon fixation and thus increase biomass production. We quantified the amount of biomass produced at the end of each tested condition. The results showed slightly different behaviors depending on the type of wastewater used as growth culture (Figure 4). In the high-reduced wastewater, we observed a 2-fold increase at –0.4 V and a 3-fold increase for the –0.8 V potential. In contrast, in the low-reduced wastewater there was a 4-fold increase at –0.4 V and a 7-fold increase in the reactor polarized at –0.8 V. These results showed that, unlike the water quality parameters (TOC and COD), biomass production was highly affected by cathodic polarization.

The impact in biomass production for both types of wastewater was consistent with PPB metabolism previously reported in presence of a high-reduced carbon source. Indeed, this condition activates the electron sinks so the microorganisms become less sensitive to cathodic polarization (McKinlay and Harwood, 2010; Vasiliadou et al., 2018; Guzman et al., 2019). This may explain why polarization had a greater impact when PPB where grown using low-reduced carbon sources.

#### 3.1.3. Stirred cathodes: High impact but low current

Despite the impact of polarization on biomass production, we observed less consumption of electrical current than expected (*ca.* −50 to −100 μA; Supplementary Figure S2). We hypothesize that the cathode could be acting as a metabolic regulatory element rather than as a true electron donor, as previously reported in PPB electrofermentation processes (Manchon et al., 2023b). Under polarization conditions where hydrogen should be produced (*ca.* −0.8 V), we monitor higher current consumption (−80 to −100 μA). We are certainly producing less hydrogen than the one expected with non-carbonaceous materials like platinized titanium; unfortunately, we doubt this material could be fluidized and still be biocompatible.

#### 3.1.4. Cathodic photo-electrofermentation leads to metabolic and population changes in the microbial community

Electrofermentation processes using microbial consortia led to changes in microbial population structure and metabolic activity. Therefore, we explored the microbial community using 16S Illumina sequencing to understand the role of polarization in microbial selection. The analyses revealed differences at all taxonomic levels although we focused on the genus level as it is the most informative regarding functionality.

**Figure 5:**
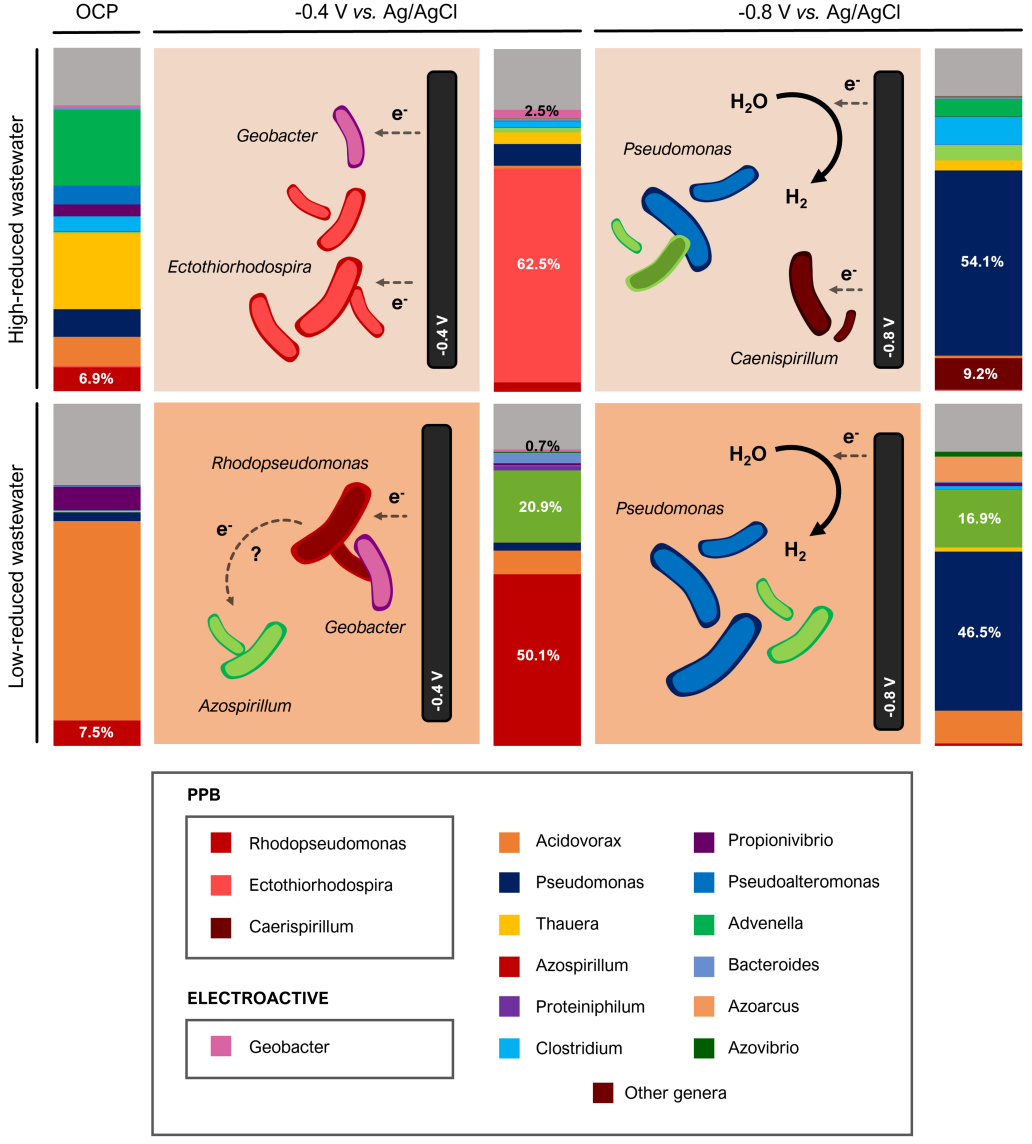
Microbial population structure at the genus level analyzed by 16S Illumina corresponding to screening with brewery wastewater.

The mere presence of electrically conductive material has been reported to favor the exchange of electrons between electroactive microorganisms even without external polarization (Rotaru et al., 2021). This phenomenon, which has been described in non-phototrophic bacteria either using fixed bed (Prado et al., 2022) or fluid-like beds (Tejedor-Sanz et al., 2018). In contrast, our stirred reactors showed similar pollutant removal efficiency and biomass production in both free-electrode reactors and stirred-electrode reactors under Open Circuit Potential (OCP; Supplementary Figures S3A,B). However, the presence of electroconductive material had an impact on the microbial population composition (Supplementary Figure S4). The electrode-free reactors showed a majority presence of microorganisms characteristic of wastewater, with *Pseudomonas* and *Azonexus* genera and a very low presence (<0.5%) of PPB genera such as *Rhodopseudomona*s or *Ectothiorhodospira* and electroactive bacteria such as *Geobacter*. On the contrary, *Rhodopseudomonas* sp., the electroactive PPB model genus (Bose et al., 2014), was present when stirred electrons were used under OCP (Figure 5). In addition, other genera such as *Pseudomonas* and *Acidovorax*, considered core genera in electroactive communities (Xiao et al., 2015), were also present. In the OCP-based reactor fed with high-reduced wastewater (5.9 e^−^/C), we found *Thauera* genus, which was described as electroactive due to its extracellular respiration of AQDS (Liu et al., 2006). In both OCP reactors, *Propionivibrio* was found, a genus also present in other electrofermentation systems, capable of producing propionic acid and of interacting with extracellular redox species (Yang et al., 2021).

Stirred-electrode reactors polarized at –0.4 V (vs. Ag/AgCl) had a completely different microbial structure, showing maximum predominance of PPB genus (Figure 5). Under polarization at this potential (−0.4 V) the production of hydrogen is thermodynamically not possible (E = −0.63 V vs. Ag/AgCl), so extracellular electron uptake was not hydrogen-mediated. Therefore, extracellular electron transfer could only occur through direct electron transfer or through self-produced redox mediators (Logan et al., 2019). This is consistent with the high presence of electroactive genera: classical electroactive genus such as *Geobacter* (Koch and Harnisch, 2016) and other electroactive PPB genera such as *Rhodopseudomonas* (Bose et al., 2014). In these reactors (−0.4 V) wastewater composition was a key factor in microbial selection (Figure 5). Thus, using high-reduced wastewater (5.9 e^−^/C), we detected a predominance of *Ectothiorhodospira* a PPB also found among electroactive communities (Manchon et al., 2023b). On the contrary, *Rhodopseudomonas*, model electroactive PPB genus (Bose et al., 2014; Guzman et al., 2019) predominated in presence of low-reduced wastewater (3.5 e^−^/C).

The microbial community analysis revealed a different scenario in the reactors polarized at –0.8 V (Figure 5). In both reactors, the analyses showed 50% *Pseudomonas* and only in presence of high-reduced wastewater fed reactor, the PPB *Caenispirillum* was observed. This population structure was, in fact, very similar to the free-electrode reactors used as control (Supplementary Figure S4). At –0.8 V, hydrogen-mediated electron transfer is feasible (E = −0.63 V vs. Ag/AgCl). Although another extracellular electron pathway (H_2_-mediated) could explain the different community observed at this potential, hydrogen does not inhibit the growth of PPB (Vasiliadou et al., 2018; Supplementary Figure S5). These results suggest that –0.8 V polarization inhibits PPB growth and although hydrogen is not involved in such inhibition, further study is needed.

### 3.2. Giving insight into fluid-like cathode photo-electrofermentation

Finally, we further study how cathodic polarization modulates PPB biomass production in a more controlled environment and sophisticated reactor. In this section, we analyzed the behavior of PPB with malate (3 e^−^/C) and acetate (4 e^−^/C) as carbon source under fluid-like cathode polarization. This experimental approach allowed us to conclude more precisely the effect of the reduction state, eliminating noise due to other parameters not controlled in the experiment with wastewater, such as the carbon-nitrogen ratio, the nature of the organic compounds, or the concentration of micronutrients. In addition, our new two-chamber electrochemical fluid-like bed reactor design allowed to study the isolated effect of the cathode on PPB metabolism, eliminating the possible effect of the counter electrode.

#### 3.2.1. Fluid cathodic electrofermentation enhances biomass production

**Figure 6:**
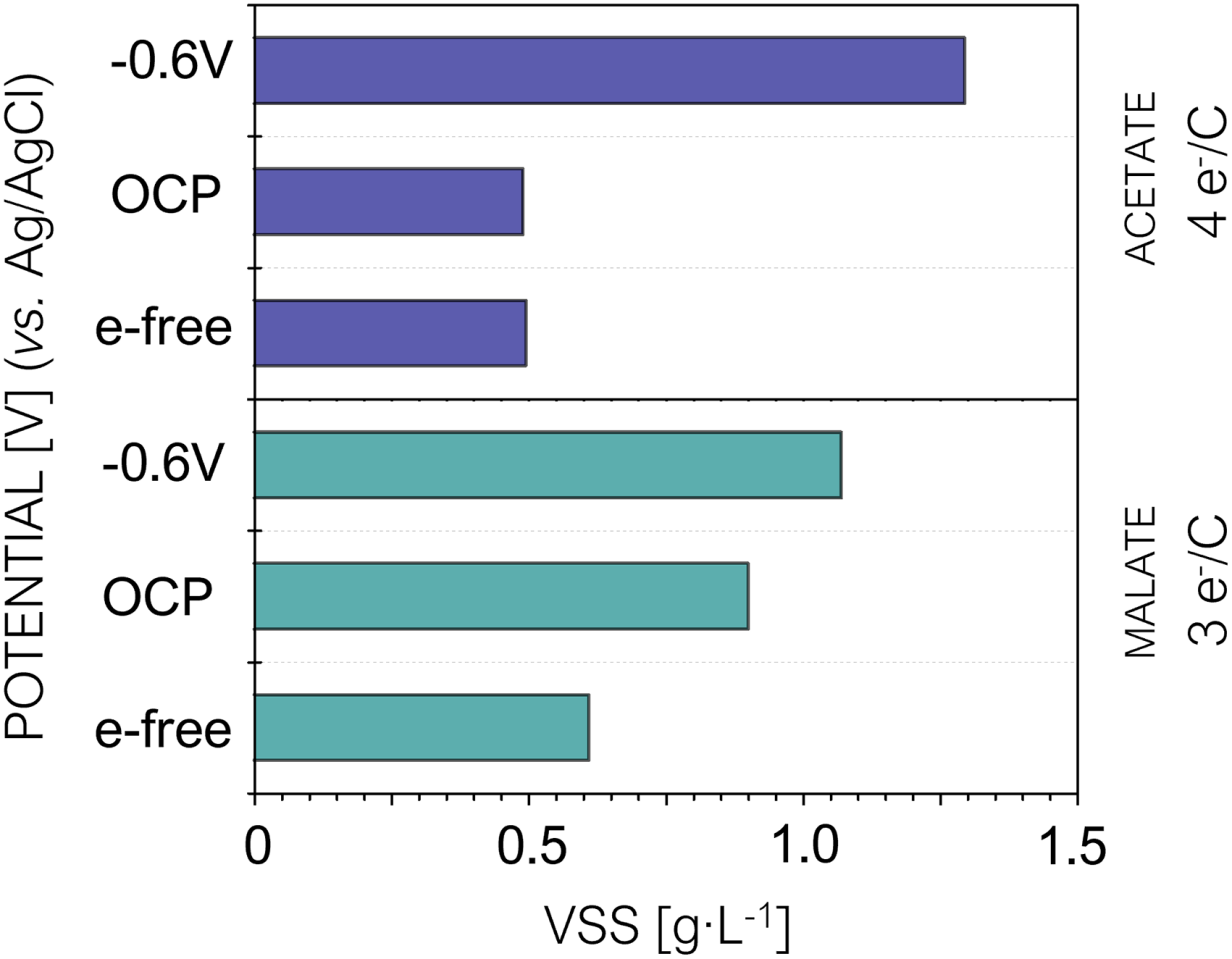
Volatile Suspended Solids (VVS) produced under electrode-free, open circuit potential (OCP) and –0.4 V (vs. Ag/AgCl).

We studied the main parameter in microbial cultivation: biomass production. Biomass analysis revealed higher production under fluid-like cathodic polarization at –0.6 V (vs. Ag/AgCl) in comparison with the electrode-free control (Figure 6). Such enhancement was confirmed for both acetate-feeding (+75%) and malate-feeding (+130%). Therefore, the results of this experiment confirmed our previous observation with stirred-electrode assays. Furthermore, they are consistent with the role of having an extra-source of electrons (cathode) on PPB metabolism, promoting carbon fixation and thus maximizing biomass production (Guzman et al., 2019).

In previous assays with stirred-electrode reactors fed with wastewater, we did not observe an impact in the biomass production (Figure 4) when the fluid-like electrode was non-polarized (OCP) in comparison with free-electrode conditions. In contrast, our fluid-like electrode (OCP) reactor fed with malate revealed higher biomass production (*ca.,* 50%) in comparison with the electrode-free control (Figure 6). This could indicate that, with oxidized carbon sources (3 e^−^/C), the electrically conductive material could specially facilitate syntrophies between microorganisms as reported elsewhere (Rotaru et al., 2014, 2018). These syntrophic relationships seem to favor microbial growth, allowing the consortium to deal more easily with redox stress situations. This hypothesis is consistent with the absence of differences in biomass production when acetate (4 e^−^/C), a more balanced electron-carbon ratio (McKinlay and Harwood, 2010) was feeding our free-electrode or OCP reactors.

#### 3.2.2. Fluid-like cathodic photo-electrofermentation promotes purple phototrophic bacteria presence

Once the effect of fluid cathode on biomass production was demonstrated, we analyzed microbial population structure by Illumina 16S sequencing. We focused on the population structure at the genus level as it is the most informative from the point of view of function and metabolism.

**Figure 7:**
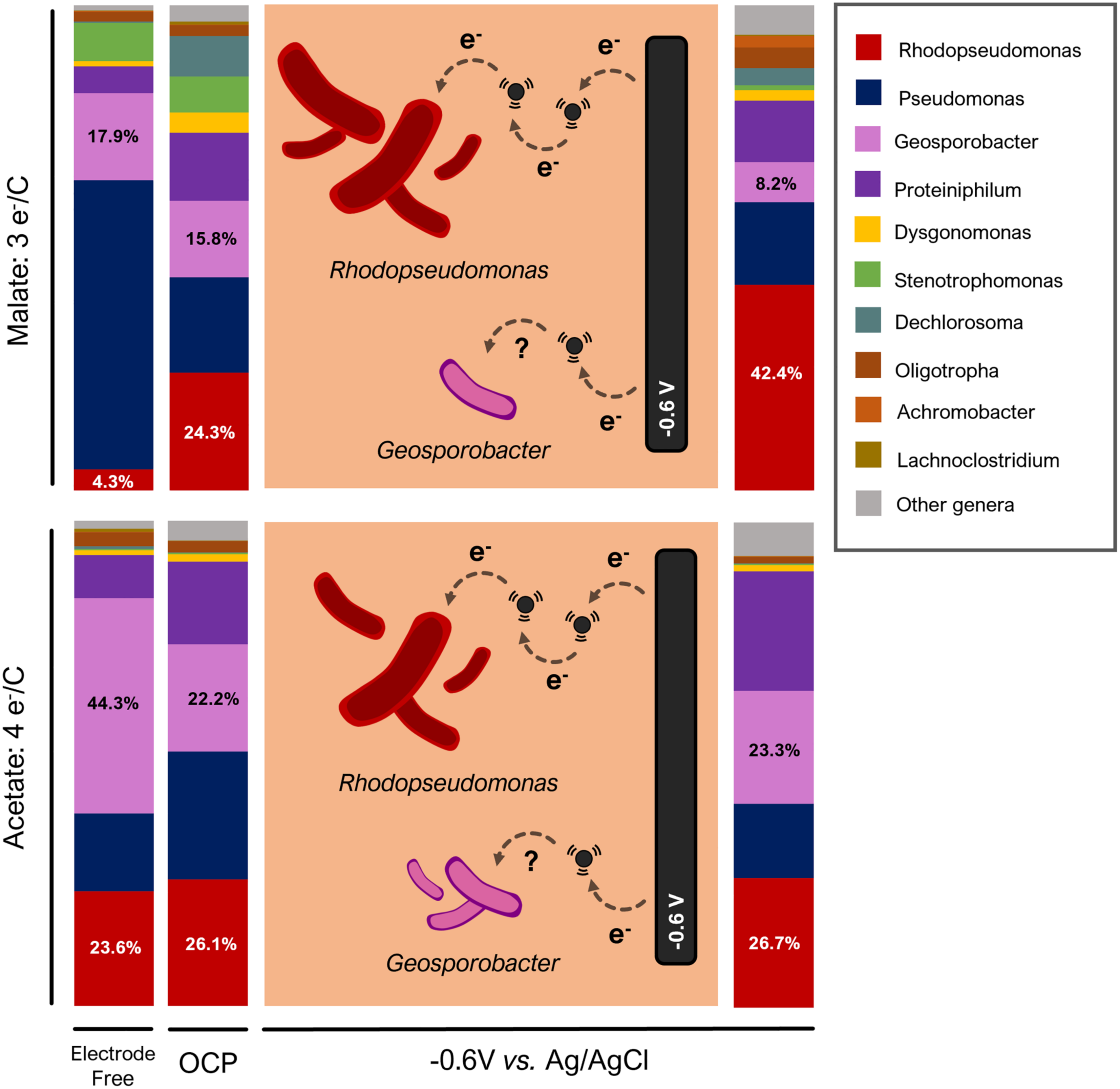
Genus-level composition (16S Illumina) of a microbial population grown with synthetic wastewater under fluid-like cathode IR-Irradiated reactor.

As we described in the assays with stirred-electrode reactors fed with wastewater (Figure 5), the mere presence of conductive material can select for a different microbial community. In this case, the differences were more evident in the malate-fed OCP reactor, with a higher relative abundance of *Rhodopseudomonas* (24.3%) compared to the free-electrode reactor (Figure 7). The acetate-fed reactor, on the other hand, showed a similar abundance of *Rhodopseudomonas* regardless the presence of a non-polarized electrode. These results were consistent with those shown in biomass production (Figure 6). Malate is a more unbalanced organic compound, so we hypothesized that the conductive material would promote electroactive bacteria from genus *Rhodopseudomonas* to deal with redox stress through electrosyntrophies (Liu et al., 2021; Rotaru et al., 2021). In the acetate-fed reactor, more redox-balanced substrate, electrically conductive material does not provide a competitive advantage to electroactive bacteria and therefore its effect was less remarkable.

Additionally, we observed a high presence of *Geosporobacter* in all the reactors, including the electrode-free control (Figure 7). Some bacteria of this genus have been described as an anaerobic iron-reducing bacteria (Hong et al., 2015) capable of colonizing electrodes (Miceli et al., 2012; Moscoviz et al., 2018). These results could indicate some type of syntrophic relationship between both genera where *Geosporobacter*, in absence of electron acceptor, may transfer electrons to *Rhodopseudomonas* which is well known for its great ability to dissipate reducing power *via* electron sinks (Guzman et al., 2019). The possible electrosyntrophy between the genera *Geosporobacter* and *Rhodopseudomonas* requires further study.

## 4. Conclusion

In this study, we have demonstrated that mobile cathodes (either stirred or fluidized) can shape planktonic microbial communities dominated by PPB. We observed that cathodic polarization and IR irradiation can play a key role in microbial and phenotypic selection, promoting the PPB activity (−0.4 V) or minimizing their presence (−0.8 V).

Furthermore, our results revealed the importance of reduction status of carbon source in the PPB photoheterotrophic culture and how electrodes drives microbial population shifts depending on the reduction status of the carbon source. Finally, we conclude that using fluid-like cathodes may accelerate the transition from the wastewater treatment model into the biorefinery model to maximize biomass production.

## Data availability statement

The raw data supporting the conclusions of this article will be made available by the authors, without undue reservation.

## Author contributions

CM, YA, and AE-N contributed to the conceptualization and design of the study. CM, YA, FM-M, ML, and ÁP contributed to the investigation and methodology. AE-N contributed to the funding acquisition, supervision, writing, and review and editing. CM contributed to the writing the original draft. All authors contributed to the article and approved the submitted version.

## Funding

This work was supported by the Spanish Ministry of Science, Innovation, and Universities—State Research Agency (AEI) and European Regional Development Fund (ERDF) through the project METFLUID—Microbial electrochemical reactors based on fluid-like electrodes: a new biotech platform for performing environmental applications. Ref. RTI2018-101974-B-C-21 (MCIU/AEI/FEDER, UE). In addition, this work was also supported by Madrid Regional Government through the project REMTAVARES. Ref: P2018/EMT-4341. CM was funded by the Industrial Ph.D. fellowship program from the Regional Government of Madrid: IND2020/AMB-17843.

## Conflict of interest

The authors declare that the research was conducted in the absence of any commercial or financial relationships that could be construed as a potential conflict of interest.

## Publisher’s note

All claims expressed in this article are solely those of the authors and do not necessarily represent those of their affiliated organizations, or those of the publisher, the editors and the reviewers. Any product that may be evaluated in this article, or claim that may be made by its manufacturer, is not guaranteed or endorsed by the publisher.
