# Supplementary material for: Fluid-like cathode enhances valuable biomass production from brewery wastewater in purple phototrophic bacteria
Source: Front Microbiol. 2023 Mar 13;14:1115956. doi: 10.3389/fmicb.2023.1115956 (PMC10040824; doi:10.3389/fmicb.2023.1115956)
Supplement: Supplementary file 1 [file Data_Sheet_1.pdf]

Supplementary Material

1 COD:TOC ratio as indicator for reduction degree in wastewater

The reduction degree of the wastewater used in the **Section 3.1** was calculated by using TOC:COD ratio.

We use standard wastewater quality analysis to assess the reduction status of organic compounds in the wastewater: the TOC allows us to assess the amount of carbon in the wastewater. The COD, on the other hand, is a good indicator of the number of electrons contained in the wastewater.

We have calculated the electrons per carbon produced in the oxidation to carbon dioxide of three carbon sources (**Table 1**), including their theoretical TOC:COD values. From the linear regression shown below, we were able to estimate the electrons per carbon of the wastewater used.

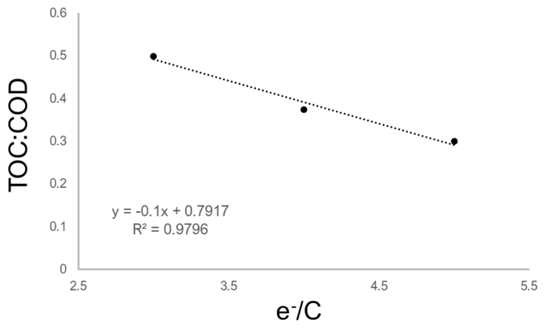

2 Supplementary figures

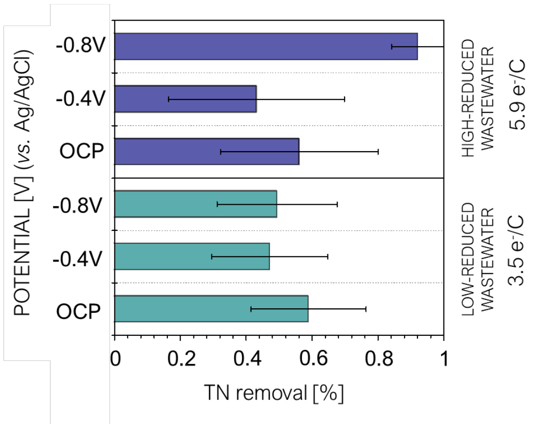

Figure 1SI. Total Nitrogen (TN) removal ± standard error under a) Open Circuit Potential (OCP),b) -0.4 V (*vs.* Ag/AgCl) , and c) -0.8V (*vs.* Ag/AgCl). HRT=5 days. Purple bars correspond to reactors fed with high-reduced wastewater and blue bars corresponds to the reactors fed with low-reduced wastewater.

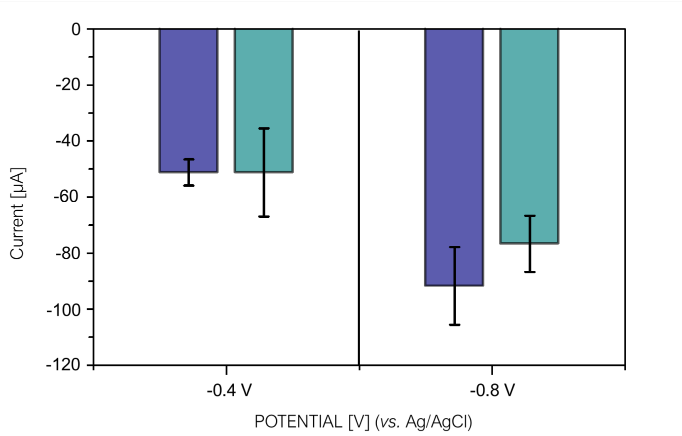

Figure 2SI. Current intensity ± standard error under a) Open Circuit Potential (OCP), b) -0.4 V (*vs.* Ag/AgCl) and c) -0.8V(*vs.* Ag/AgCl). HRT=5 days. Purple bars correspond to reactors fed with high-reduced wastewater and blue bars corresponds to the reactors fed with low-reduced wastewater.

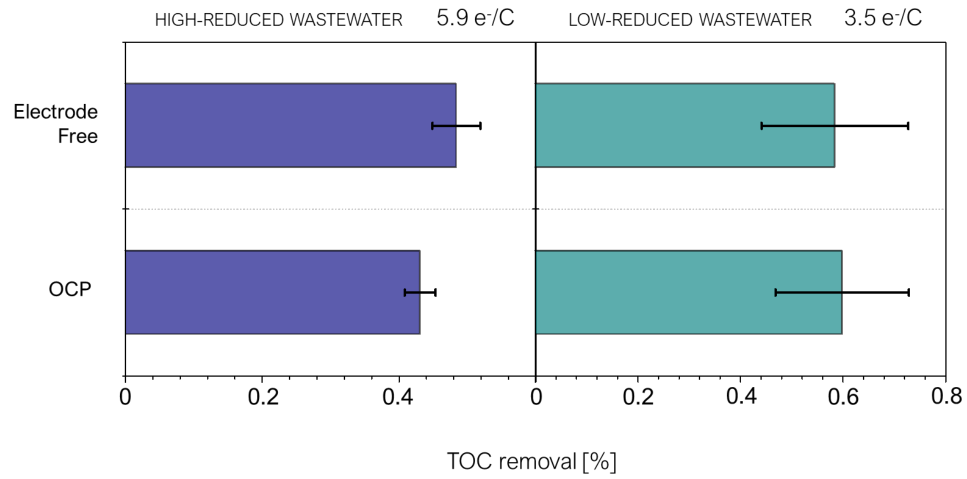

Figure 3SIa. Total Organic Carbon (TOC) removal ± standard error under a) Open Circuit Potential (OCP) and electrode-free. HRT=5 days. Purple bars corresponds to reactors fed with high-reduced wastewater and blue bars corresponds to the reactors fed with low-reduced wastewater.

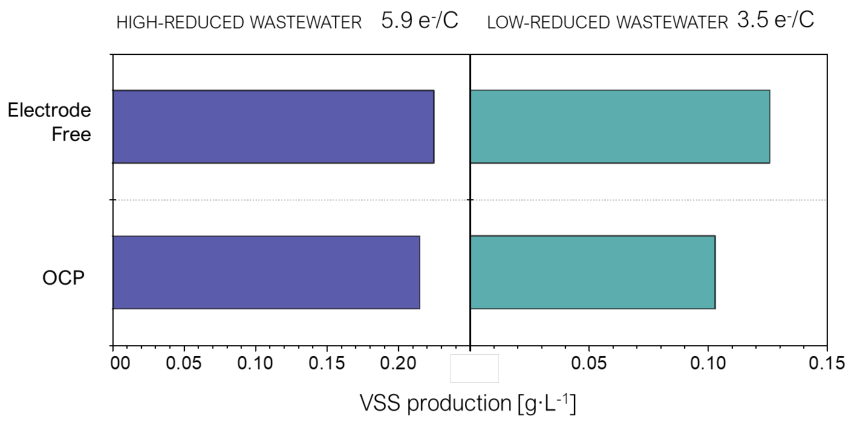

Figure 3SIb. Volatile Suspended Solids (VVS) produced at the end of the experiment under Open Circuit Potential (OCP) and electrode-free. HRT=5 days. Purple bars correspond to reactors fed with high-reduced wastewater and blue bars corresponds to the reactors fed with low-reduced wastewater.

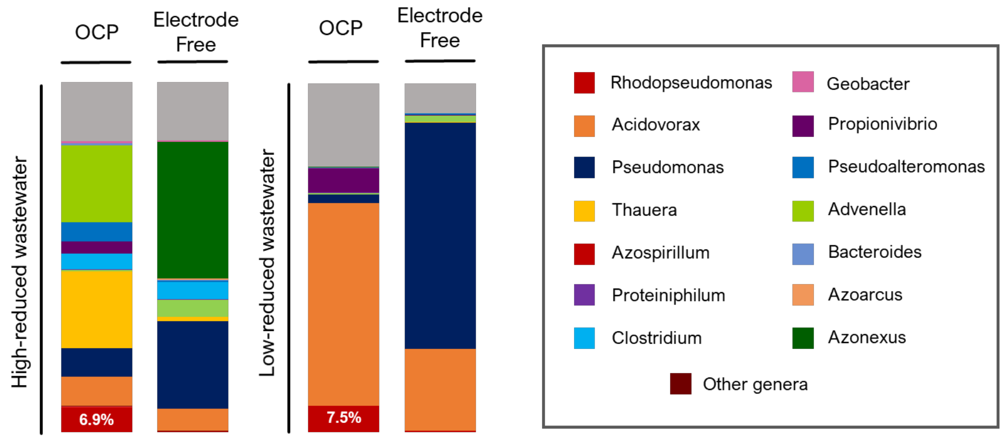

Figure 4SI. Microbial population composition at the genus level analyzed by 16S Illumina corresponding to screening with brewery wastewater (OCP and electrode-free).

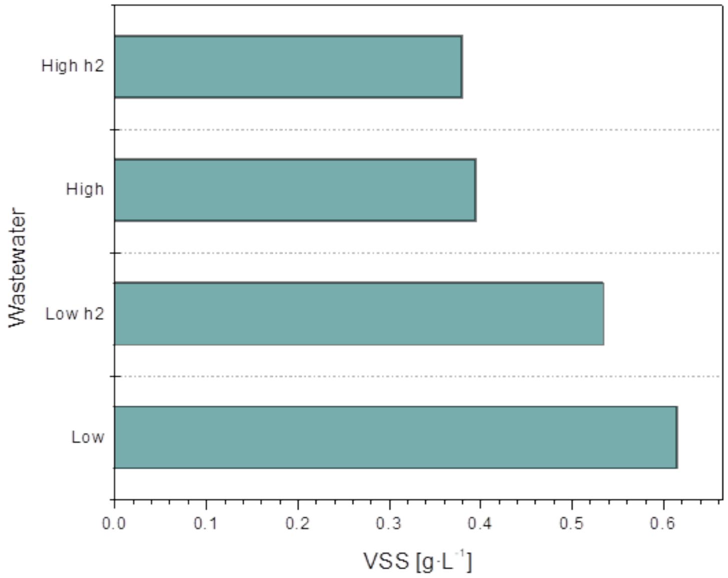

Figure 5SI. Volatile Suspended Solids (VVS) produced at the end of the experiment under -0.8 V (*vs.* Ag/AgCl) with and without hydrogen. HRT=5 days. Purple bars correspond to reactors fed with high-reduced wastewater and blue bars corresponds to the reactors fed with low-reduced wastewater.
